# Comparative Evaluation of the Essential Oil of the New Ukrainian *Lavandula angustifolia* and *Lavandula x intermedia* Cultivars Grown on the Same Plots

**DOI:** 10.3390/molecules27072152

**Published:** 2022-03-26

**Authors:** Katarzyna Pokajewicz, Marietta Białoń, Liudmyla Svydenko, Nataliia Hudz, Radosław Balwierz, Dominik Marciniak, Piotr Paweł Wieczorek

**Affiliations:** 1 Department of Analytical Chemistry, University of Opole, 45-052 Opole, Poland; marietta.bialon@uni.opole.pl (M.B.); pwiecz@uni.opole.pl (P.P.W.); 2 Sector of Mobilization and Conservation of Plant Resources, Rice Institute, National Academy of Agrarian Sciences, 74992 Kherson, Ukraine; svid65@ukr.net; 3 Department of Drug Technology and Biopharmaceutics, Danylo Halytsky Lviv National Medical University, 79010 Lviv, Ukraine; natali_gudz@ukr.net; 4 Department of Pharmacy and Ecological Chemistry, University of Opole, 45-052 Opole, Poland; radoslaw.balwierz@uni.opole.pl; 5 Department of Drug Forms Technology, Faculty of Pharmacy, Wrocław Medical University, 50-556 Wroclaw, Poland; dominik.marciniak@umw.edu.pl

**Keywords:** *Lavandula*, lavandin, lavender, essential oil, linalool, composition, GC–MS, caryophyllene oxide

## Abstract

New cultivars of lavender adapted to arid steppe conditions were developed by the Institute of Rice of Ukrainian National Academy of Agrarian Sciences (NAAS). This work is a part of the characterization process of the new cultivars. The chemical composition of the essential oil of the seven new *Lavandula angustifolia* and eight new *Lavandula x intermedia* cultivars was investigated and compared. In total, 71 different compounds were identified. Linalool and linalool acetate were the main components in both species in ranges of 26.14–57.07% and 9.08–24.45%, respectively. They were followed by terpinen-4-ol (2.16–22.44%), lavandulyl acetate (2.12–10.23%), and lavandulol (1.30–3.14) in the case of *L. angustifolia* and camphor (10.11–12.55%), borneol (5.49–8.71%), and eucalyptol (0.47–7.41%) in the case of *L. x intermedia*. The oils had a valuable terpene profile—a high linalool content and the substantial presence of lavandulol and its ester. Nevertheless, they did not comply with the industry standards, mostly due to high levels of terpinene-4-ol. Evidently, a high content of terpinen-4-ol is a characteristic feature of *L. angustifolia* oils bred in Ukraine. Additionally, the LA3 cultivar yielded an oil with some of the highest linalool contents reported in the literature. Statistical analysis and literature data allowed for the comparative analysis of the gathered data. MANOVA, PCA, and HCA marked caryophyllene oxide as another potential differentiating compound between studied species.

## 1. Introduction

Lavender is a group of aromatic shrub plants originating from the Mediterranean basin [1,2]. Its name originates from the Latin word “lavare”, which means “to wash” or “to bath”, as it was used for washing by the ancient Arabians, Greeks, and Romans [3,4]. Lavender has a long history of use in traditional medicine, and its oil is used topically, orally, or inhaled for multiple conditions due to its presumed disinfecting, carminative, cicatrizing, calming, and sedative activities [4]. Today, apart from the traditional medicinal use, lavender oils are employed for scenting cosmetic and perfume products, as well as in food flavoring. The plant is still gaining popularity, and its cultivation area and essential oil production are growing [1,3]. Lavender is cultivated in many countries around the world, especially in Bulgaria and France.

The genus *Lavandula* belongs to the family Lamiaceae and contains 39 species and eight hybrids [2,3,5]. The common name lavender is usually applied to several species of *Lavandula* generally used for their aromatic quality [1]. We can distinguish such species as *L angustifolia*, *L. latifolia*, *L. dentata*, *L. stoechas*, and *L. x intermedia*. The main species used for distilling essential oil are *L. angustifolia*, *L. latifolia,* and *L. x intermedia*, which is an interspecies hybrid of the first two species.

*L. angustifolia* (LA) Miller is also referred to as true lavender or English lavender that is used for producing the most valuable essential oil known as true lavender oil or just lavender oil.

*L. latifolia* (LL) Medikus is also known as spike lavender, cultivated mostly in Spain, and used as the raw material for obtaining spike oil.

*L. x intermedia* (LI) Emeric ex Loiseleur, also known as lavandin, Dutch lavender, and *Lavandula hybrida,* is a sterile hybrid between spike and English lavender. It is a much larger plant than *L. angustifolia* and is much appreciated for its high yield of essential oil.

True lavender oil is recognized as being the best in terms of quality because it contains a lower camphor content than spike or lavandin oil. Therefore, true lavender essential oils are mostly used in the perfume industry, while other oils are mostly used in detergents and cosmetics products [6,7]. Lavandin plants produce more oil and are hardier than lavender plants [1,7,8]. True lavender and lavandin yield from 8 to 30 kg and from 40 to 220 kg, respectively, of essential oil per hectare depending on cultivar, location, and conditions [8]. Hence, prices of true lavender oil are 3–7 times higher than those of lavandin [1,9]. Spike oil is also much cheaper. However, its flavor and chemical composition differ more from true lavender oil than lavandin oil. Thus, lavandin is often the preferred species for cultivation, and the worldwide production of lavandin oil is currently a few times larger than of true lavender oil [1]. Since pure lavender oil is more expensive and is subjected to periodical supply shortages, lavandin oil is often used as replacement oil or an adulterant to counterfeit true lavender oils [10].

The pleasant aroma of lavender plants is caused by the presence of the volatile terpenoid compounds (mainly monoterpenoids) that are synthesized and accumulated in aerial parts, mostly flowers [2]. These volatile constituents are isolated from plants during the hydrodistillation process to obtain essential oils. Lavender essential oils contain mainly oxygenated monoterpenes such as linalool, linalyl acetate, eucalyptol (1,8-cineole), terpineols, camphor, borneol, and unique irregular oxygenated monoterpenes such as lavandulol and lavandulyl acetate. The other terpene groups such as monoterpene hydrocarbons (*β*-ocimene, *α*- and *β*-pinene), sesquiterpene hydrocarbons (*β*-farnesene and caryophyllene), and oxygenated sesquiterpenes (caryophyllene oxide) constitute a lesser fraction of lavender oils [2]. Commonly, the main components are linalool and its acetyl ester, linalyl acetate. According to a review conducted by Aprotosoaie et al. [11] on the chemical composition of different lavender essential oils, these components constitute 1–54 and 4–57% of true lavender oil and 2–48 and 2–48% of lavandin oil. However, the lower limits of these components are less common, and the concentration is usually over 20%. The presence of higher amounts of linalool and linalyl acetate is desirable because it leads to the better floral aromatic and pharmaceutical quality of the oils [11,12]. Therefore, the oils are often adulterated with synthetic linalool and linalyl acetate. Other significant lavender EO (essential oil) components include terpinen-4-ol, *α*-terpineol, lavandulol, lavandulyl acetate, *β*-ocimene, eucalyptol, and borneol. Regarding the first two terpineols, their content in true lavender oil is 2–14 and 2–9% and 0.4–16% and 2–10% in lavandin oil, respectively [11,13]. Terpinen-4-ol is usually more abundant in LA oils than *α*-terpineol. Lavandulol and lavandulyl acetate are distinctive to lavender plants and beneficial, and their high concentration provides a characteristic herbal-rosy scent; on the other hand, the oil aroma is adversely affected by ocimene, eucalyptol, and camphor [14]. *β*-ocimene may be present in true lavender EOs at levels of 2–18% and 0.5–6% for *cis*- and *trans*-isomers, respectively, and in lavandin oil at levels of 2–15% and 0.2–9% for *cis*- and *trans*-isomers, respectively [11,15,16,17]. High levels of eucalyptol and camphor may also be present in LA oil (from 0.1 to 44 and from traces to 28%, respectively) but the mentioned high values predominate in the essential oils from lavender leaves and stems or flowers from some unique special chemotypes [11,18,19]. Usually, the oils obtained from flowering tops of *L. angustifolia* contain low levels of these compounds—from traces up to 2%. In contrast, lavandin oils commonly exhibit higher contents of eucalyptol and camphor (2–49 and 2–33%, respectively). Though not illustrated by the latter ranges due to high variability, an assessment of different results gathered in tables by Aprotosoaie et al. [11] showed median values at about 7% (for both components). On the one hand, much higher eucalyptol/camphor contents in *L. x intermedia* EOs result in a lower oil quality for the perfumery industry. On the other hand, this oil is more effective as an antimicrobial agent [17,20]. Overall, lavender/lavandin oils can contain over a hundred different volatile oil components [11]. However, we need to be aware that some of the identified compounds may come from the degradation that occurs during the hydrodistillation process [2].

A literature overview of studies on lavender and lavandin oils demonstrates huge variability in their chemical composition. Intraspecific differences are caused by different cultivars, geographical locations, weather, soil type, harvesting time, the processing of the plant material, and its extraction method [11,12,21,22]. The complexity and changeability of essential oils, not mentioning the possibility of their adulteration, has led to greater pains caused by the problem of market-available lavender oils variance. It is difficult to consider the topic of lavender EOs, to conduct replicable research on their biological activities, or even to manufacture products of consistent quality. To help the industry cope with this issue, some important standards have been established regarding the chemical composition of lavender essential oils: (1) ISO 3515:2002, which covers six norms for *L. angustifolia* essential oils of six different origins; (2) ISO 8902:2009, which covers lavandin ‘Grosso’ EO; (3) ISO 3054:2001, which covers lavandin ‘Abrial’ EO; and (4) ISO 4719:2012, which covers spike lavender and the European Pharmacopeia (Ph. Eur.) monograph for *L. angustifolia* EO. These standards are presented in Table 1.

As stated before, the market of lavender products is growing and many countries are increasing their numbers of farms and production areas [1]. In Ukraine, before 2014, the plantations of essential oil crops were located in the territory of the Autonomous Republic of Crimea. After the Crimea annexation, there was a need to introduce these crops to the other regions of the country. Therefore, the sector of the mobilization and conservation of plant resources at the Institute of Rice of National Academy of Agrarian Sciences (NAAS) was assigned with the task GDR24.01.01.32. P.: “Formation of collections of aromatic plants for the development of the varieties adapted to the steppe zone of southern Ukraine”. The Institute works on the breeding and selection of new cultivars of *L. angustifolia* and *L. x intermedia* that are of high economic value and are adapted to the arid conditions of the southern steppe of Ukraine [23]. The new cultivars have been studied in many ways. The goal of this research work was to characterize the chemical compositions of the EOs of the new cultivars developed by the Institute of Rice of NAAS. The findings supplement knowledge about new cultivars, enabling choices of plants with valuable features for mass cultivation as well as the selection of specimens for further breeding. Additionally, we compared the composition of *L. angustifolia* and *L. x intermedia* EOs from plants growing on the same plots to show interspecies differences and discuss the results in reference to previous findings. In the second part of the study, we compiled new results with our previous ones [18] and employed statistical tools for a broader comparative analysis.

## 2. Results

### 2.1. Part A—New Results for L. angustifolia and L. x intermedia EOs

The chemical composition of essential oils from seven new cultivars of *L. angustifolia* and eight new cultivars of *L. x intermedia* developed by the Rice Institute of the NAAS was analyzed. Due to a large amount of data, the whole dataset including standard deviations is only presented in Supplementary Materials (Table S1). Table 2 presents the components detected in the *L. angustifolia* EOs samples. All of them were obtained in 2020, most at the total flowering phase, except for cultivar LA2, which was sampled both at the total flowering and the end of flowering phase. We identified or tentatively identified 71 different compounds in these oils.

**Figure 1:**
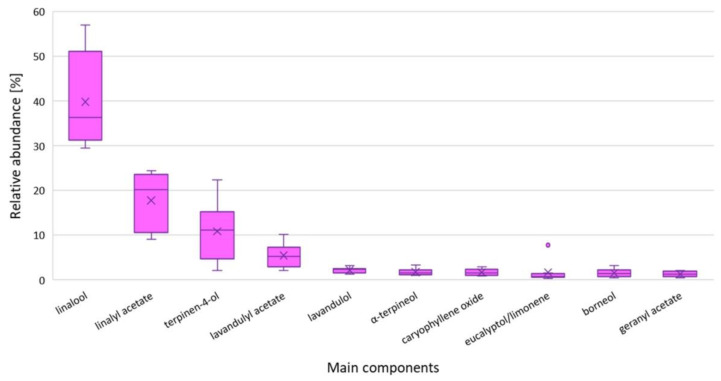
The relative abundances of the main components in the studied *L. angustifolia* essential oils. The boxes represent the Q1–Q3 interquartile ranges. Lines intersecting the box represent the medians, and crosses represent the average value. Whiskers represent the lowest and highest results. Dots represent outlying results.

The main components were linalool (29.51–57.07%) and linalyl acetate (9.08–24.45%), which is typical for *L*. *angustifolia* EOs. These compounds together constituted from 53 to 68% of oil, with a predominance of linalool (Figure 1). Two cultivars, LA2 and LA3, were characterized by an especially high amount of linalool: 55.08 and 57.07%, respectively. In fact, the latter is one of the highest values reported in the literature [11,19]. The analysis of the oil obtained from the LA2 lavender cultivar showed that the composition of the oil changed with the maturation stage of the flower. The linalool content was much higher and the content of its ester was lower. This is in line with other findings showing that the lavender flowering stage is the important factor affecting monoterpenols and their ester production, as well as showing that the later flowering stages are beneficial for linalool production [11,26]. Comparing the results of these seven new LA cultivars to the results obtained for other cultivars developed in the Rice Institute and described in previous work [18], we can conclude that the linalool content was higher and its acetate content was lower. This may have been caused by intercultivar variations and/or the summer of 2020 with little rainfall and lesser soil moisture compared to 2016–2020 in the Kherson region.

The content of terpinen-4-ol in the studied oils was remarkably high for true lavender. It ranged from 2.16 to 22.44%, with a median of around 11%. Undeniably, six of eight samples contained more than 10% of this monoterpenol. Different authors have reported values in a range from 0.11 to 18.7%, but most of them have been close to 3%, rarely reaching 7% or higher [11,15,18,19,27]. Similarly high levels of terpinen-4-ol were previously observed by us in the aforementioned lavender EOs of Ukrainian origin, where 7 out of 13 cultivars were characterized by a content of 10% or higher [18]. The Crimean true lavender oil characterized by Białoń et al. contained about 7% of this component. The significant concentrations of terpinen-4-ol seem to be a characteristic feature of lavender EOs of Ukrainian origin.

The other significant components in the studied true lavender oils were lavandulyl acetate (2.12–10.23%), lavandulol (1.30–3.14%), and *α*-terpineol (0.94–3.37%). Table 3 presents the ten main components of the analyzed oils. The amount of eucalyptol was not large, which is preferential for EO properties, except for the LA4 cultivar, where it constituted 7.79% of the sample (accurate value not known due to coelution with limonene present in small quantities). Despite a large variability of values found in literature for this component (0.1–44%), most true lavender oils contain lower levels of these compounds from traces up to 2% [11,18,19].

Despite the fact that most of the analyzed oils presented desirable chemical characteristics expected from true lavenders oils such as a high linalool content, the significant presence of lavandulol and its ester, and mostly minor contents of eucalyptol and camphor, the oils were found to not comply with Ph. Eur. and ISO 3515 (other origins) norms. Supplementary Tables S2 and S3 show comparative tables with % relative abundances and normative content values given by these standards. The main reasons for the ISO nonconformity were the too-low contents of linalyl acetate and *β*-ocimene and, in some cases, the too-high contents of linalool and terpinen-4-ol. Likewise from a pharmacopeial point of view, the oils were found to be non-compliant mainly in terms of too-low and too-high linalyl acetate and terpineols contents, respectively. This is in line with the results obtained for other LA cultivars obtained in the same Institute [18].

The samples of EOs from eight different cultivars of *L. x intermedia* were acquired during the total flowering stage in years 2016–2020. Their chemical composition was studied with GC–MS, and Table 4 presents the results of this investigation.

**Figure 2:**
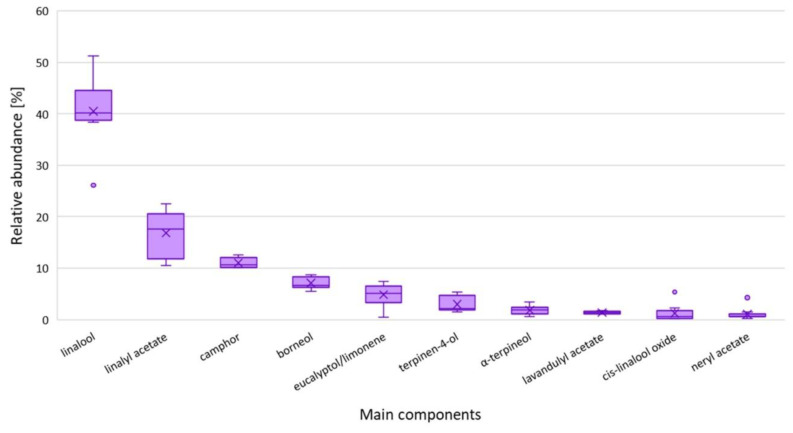
Box plots for relative abundancies of main components in the studied *L. x intermedia* essential oils. The boxes represent the Q1–Q3 interquartile ranges. Lines intersecting the box represent the medians, and crosses represent the average value. Whiskers represent the lowest and highest results. Dots represent outlying results.

In total, 61 components were identified in the studied lavandin oils. Similarly to true lavender oil, the main constituents were linalool followed by linalyl acetate (Table 5). The major component was present in the range of 26.14–51.24%, but the lower range was outlying and such a low linalool content was only present in one cultivar—LI2. Figure 2 indicates that most results were between 39 and 45%. This is in line with the values reported in the literature 1.7–62.7% [11,13] but a little higher than their average, with a range of 25–40%. The linalool content in the studied lavender oil was higher than existing ISO norms for lavandin oils and close to ISO spike oil specifications [28,29,30]. Unlike *L. angustifolia*, there is no general ISO norm for essential oil from *L. x intermedia*, only for the ‘Abrial’ and ‘Grosso’ cultivars. The normative specifications for them and comparisons to our studied samples are given in Table 6.

**Table 5:** The top ten abundant components in essential oils of the studied lavandin cultivars. The compounds are ordered according to the total sum of % area for all the cultivars. Numbers are % abundances. A heatmap of all components is presented in Figure 3.

Rank	Component	Cultivar								
		LI1	LI2	LI3		LI4	LI5	LI6	LI7	LI8
		2016	2016	2017	2018	2020	2020	2020	2020	2020
1	linalool	40.16	26.14	39.85	42.93	38.38	39.15	51.24	46.21	40.17
2	linalyl acetate	17.55	11.51	16.50	19.67	19.86	22.47	10.57	12.10	21.23
3	camphor	10.11	10.13	12.55	10.21	12.27	10.32	10.61	11.32	11.91
4	borneol	6.31	8.69	8.71	7.86	6.63	6.20	5.49	7.33	6.42
5	eucalyptol + limonene (small)	7.41	0.47	6.24	2.65	5.00	5.09	6.01	3.98	6.71
6	terpinen-4-ol	4.37	2.65	2.12	1.85	1.86	1.80	5.35	4.94	1.54
7	*α*-terpineol	1.14	0.58	2.00	3.42	2.73	1.98	1.15	1.25	1.83
8	lavandulyl acetate	1.12	1.14	1.15	1.47	1.80	1.81	1.15	1.31	1.44
9	*cis*-linalool oxide	2.21	5.35	1.20	0.67	0.20	0.14	0.21	0.54	0.42
10	neryl acetate	1.43	4.25	0.80	0.72	0.57	0.60	0.15	0.59	0.51
11	lavandulol	0.60	1.57	0.98	0.93	0.89	0.65	1.26	1.43	0.59
12	hexyl butanoate + 2		2.24		0.91	0.70	0.74	0.63	0.91	0.72
13	*cis*-sabinene hydrate	0.33	5.66	0.12		0.08	0.11	0.20	0.18	0.14
14	geranyl acetate	0.47	0.54	0.57	1.33	0.98	1.10	0.25	0.75	0.71
15	*trans*-linalool oxide	2.07		1.14	0.72	0.27	0.21	0.28	0.67	0.51

The second main component, linalyl acetate, was present in a range from 10.57 to 22.47%, with a median of 17.55%. This was lower than the ISO norm specification for ‘Abrial’ and much lower than that for ‘Grosso’, which is the French lavandin that contains high concentrations of linalool and linalyl acetate. The literature review indicates reported linalyl acetate values between 1.5 and 48%, with an average of around 25% [11]. Thus, the studied samples were characterized by a lower-than-usual linalyl acetate content. This could be due to the characteristics of the new cultivars, cultivation place, and/or weather conditions [11]. In 2017 and 2018, Detar et al. observed extremely low concentrations of linalyl acetate (3.4 and 5.3%, respectively) for the oils from the cultivar ‘Grosso’ cultivated in Hungary and harvested at full bloom stage. They assumed that this was an effect of special growth location and pedoclimatic conditions [15]. However, in 2021, they published another paper describing the influence of the flowering stage on the oil composition, where they grew the same ‘Grosso’ cultivar on Hungarian territory and in the same year of 2018 but in a different location with a different soil type. Interestingly, linalyl acetate content was also low, though only for the green bud stage. For more mature flowering stages, the content was around 20% [13]. Therefore, it is difficult to judge the determining factor, and oil composition is the result of plant genotype and many known and unknown factors. We need also to keep in mind that linalyl acetate is hydrolyzed in considerable amounts during prolonged steam distillation [31]. In our investigation, the plots, flowering stages, and distillation times were the same, and we did not observe the clear influence of years on the linalool/linalyl acetate ratio. The ratio was on average 2.4 and varied depending on the cultivar (from 1.7 to 4.9%).

The next three main constituents of the studied lavandin oils were camphor, borneol, and eucalyptol. These components are characteristic of lavandin oil, as they are present on higher levels than in true lavender oils. Camphor was present in all the tested samples at a level of 11% (with little spread; see Figure 2). This level is typical for *L. x intermedia*, where the reported range is 2–33% [11], but it is slightly higher than acceptable for the ‘Abrial” and “Grosso” ISO specifications. In terms of this compound, the investigated oils better fit the *L. latifolia* Spanish-type ISO 4719 specification. Another compound, borneol, constituted around 7% of the studied oil, which was much larger than in the previously mentioned standards and values reported for lavandin oils of French origin. Nevertheless, multiple authors have reported similar or higher values, e.g., 6.4–8.2% for “Grosso” and 5.8–6.9% for ‘Grappenhall’ cultivated in Hungary, 7.1% for lavandin of Romanian origin or even higher in the case of Macedonian (13.7–16.7%) and Iranian origin (17.1–26.4%) [11,13,32]. The investigated oils contained on average 5% of eucalyptol (the exact value is not given due to coelution with limonene). This estimation shows that the eucalyptol content was similar to that for “Grosso” oils, lower than for ‘Abrial’, and much less than that for spike oil. The content was also a little below the median of literature reported values for *L. x intermedia* (based on [11]). The other top constituents in the investigated oils were terpineols: terpinen-4-ol (1.54–5.35%, with a median of 2.12%) and *α*-terpineol (0.58–3.42%, with a median of 1.83%). These content values were higher than ISO standards but similar or even lower than many results published by other researchers [11,13]. All the other main or regulated components that were not described above are presented in Table 5 and Table 6.

**Figure 4:**
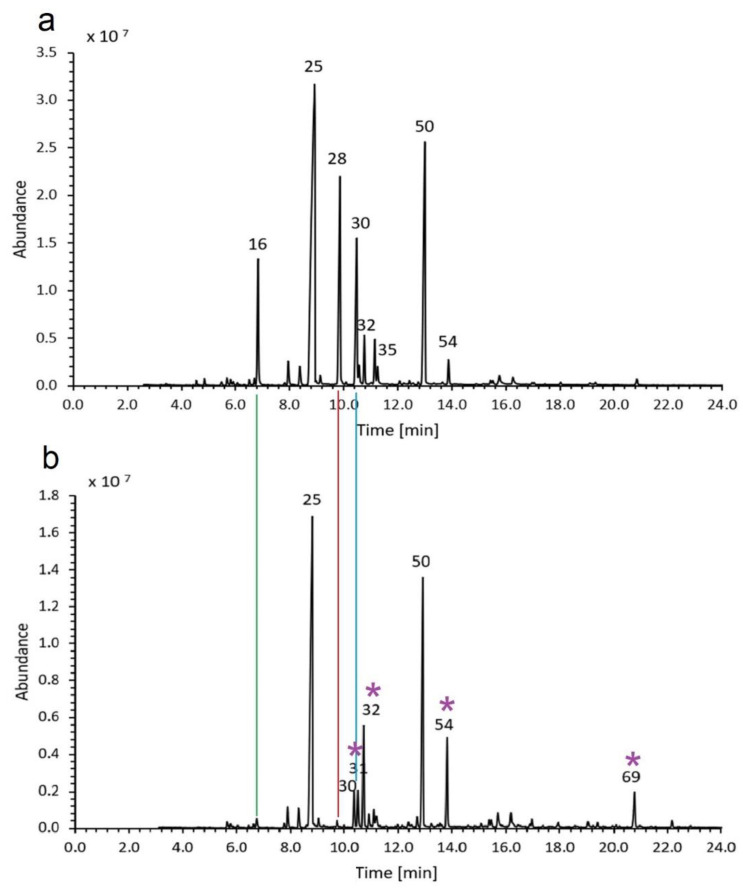
Exemplary chromatograms of lavandin ((**a**), sample ID 226) and true lavender ((**b**), sample ID 452) essential oil. The peaks were numbered according to Table 2 and Table 4. The lines illustrate which components were more abundant in lavandin oil: eucalyptol (green), camphor (red), and borneol (blue). The asterisks highlight which components were more abundant in true lavender oils (lavandulol, terpinen-4-ol, lavandulyl acetate, and caryophyllene oxide).

Comparing the studied EOs from the new cultivars of *L. x intermedia* and *L. angustifolia* grown on the same plots to each other, we could observe a much higher content of camphor (11.0 vs. 0.6%, respectively), borneol (7.1 vs. 1.5%, respectively), and (to a lesser extent) eucalyptol in lavandin oil. These are typical differences between those two species. The amount of the following compounds was lower in lavandin oils than in true lavender oils: terpinen-4-ol, lavandulyl acetate, lavandulol and caryophyllene oxide. The differences were large enough to produce different peak patterns on chromatograms. Figure 4 shows exemplary chromatograms for LA and LI essential oil samples, and it highlights the component differences noticed for our samples. The contents of the main component, linalool, and its acetate ester were similar. The same trend was observed for *α*-terpineol, linalool oxide, neryl acetate, myrcene, and ocimene (Table 7).

This study adds to the knowledge of the new cultivars bred by Rice Institute. In addition to morphological features, we could supplement cultivars’ characteristics with the oil yield and chemical composition of the essential oil. The features of the studied cultivars are presented in Table 8. It is important to note that two of the above-described cultivars, LA2 and LA7, are already sold in Ukraine.

### 2.2. Part B—Compilation of Current and Previous Results—Statistical Analysis

In this part of this study, we compare the presented above results for cultivars LA1-LA7 of *L. angustifolia* and LI1-LI8 of *L. x intermedia* with our previously published results for 15 other cultivars of *L. angustifolia* (marked here as A1–A15) [18]. All the tested samples were obtained from plants grown on the same experimental plot and were analyzed using the same analytical method and instrument. Gathering such a unique big dataset (for 27 cultivars in total) was a good opportunity to investigate deeper the correlation of EO components content with different factors such as species, cultivars, and year. Therefore, all the mentioned data were further investigated using more advanced statistical methods.

**Figure 5:**
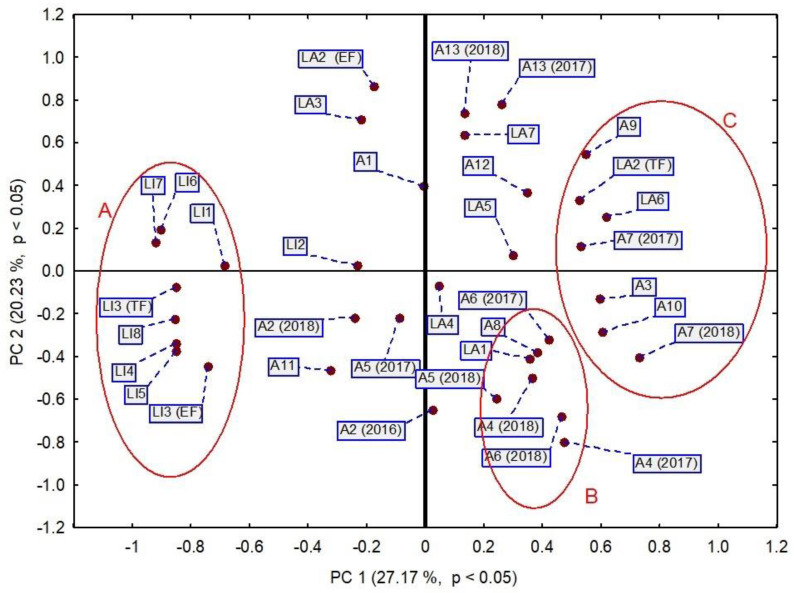
PCA score plot of all obtained data based on average content of EO components in different cultivars. PC—principal component.

Firstly, a Spearman correlation matrix analysis was conducted. The results are presented in Supplementary Table S4. This matrix showed little differentiation between the analyzed variables. Almost all the correlation coefficients between cultivars had a value of 0.7 or more, which indicated a fairly strong or very strong relationship. That means that the EO compositions of all the tested cultivars were generally similar. Therefore, principal component analysis (PCA) and hierarchical cluster analysis (HCA) using Euclidean distances were applied to order the cultivars. The results of the first analysis are shown in Figure 5. The first two principal components (PCs) explained 47.4% of the variance in the data (27.17% for PC1 and 20.23% for PC2). PCA analysis allowed us to differentiate three main groups of cultivars. The first homogeneous group (cluster A) was composed of *Lavandula x intermedia* cultivars, except for LI2. Cultivar LI2 was characterized by the lowest contents of linalool and α-terpineol and the highest content of lavandulol among all *L. intermedia* cultivars (LI1-8). It was also found to contain extraordinary high levels of *cis*-sabinene hydrate and *cis*-linalool oxide. It was distinct and did not match other PCA cluster groups. The remaining LI cultivars were quite similar to each other in terms of essential oil composition. Cultivars LI1, LI6, and LI7 positively contributed to the analysis, as they were characterized by similar contents of camphor (10.11–11.32%), borneol (5.49–7.33%), α-terpineol (1.14–1.25%), and lavandulyl acetate (1.12–1.31%). The entire cluster A containing almost all LI cultivars was significantly different from the rest of the data. This confirms the fact that the composition of lavandin essential oil is different than that of true lavender oil.

The remaining clusters, B and C, only contained cultivars of *L. angustifolia*. Cluster B consisted of cultivars LA1, A4, A5 (2018), A6, and A8, with similar contents of compounds *β*-myrcene (0.14–0.40%), *p*-cymene (0.17–0.39%), and lavandulol (0.35–1.36%). The third group (cluster C) consisted of LA2 (TF) and LA6, as well as A3, A7, A9, and A10, with comparable values of linalool (19.8–35.4%), camphor (0.22–0.98%), 3-octanone (0.26–0.95%), and the sum of butyl butanoate and 3-octanol (0.2–0.6%). The differentiating component between group B and C was the terpinen-4-ol content, which was clearly higher in group C than in group B. In group B, the terpinen-4-ol abundancies ranged from 1.17 to 10.73%, and in group C, they ranged from 6.52 to 18.7%. The other cultivars represented by the points close to the center of the plot did not belong to the described above clusters.

**Figure 6:**
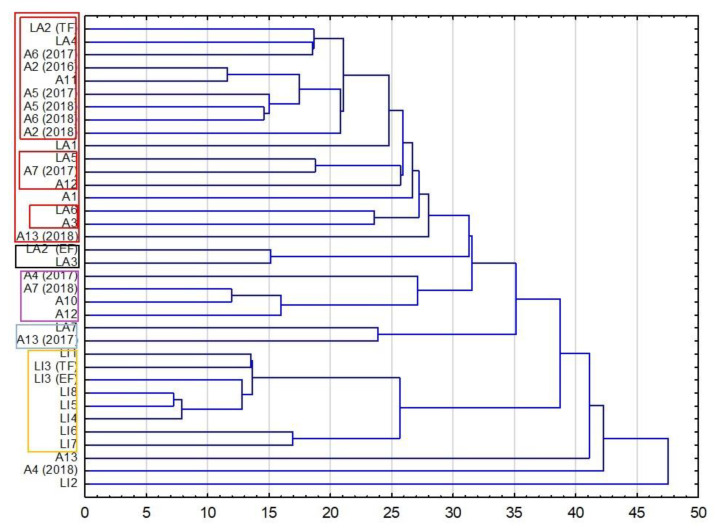
Dendrogram obtained via the HCA analysis of all obtained data based on the average content of EO components in different cultivars.

The conclusions from the PCA analysis, which was based on the comparison of the correlation coefficients (the experiment matrix was the covariance matrix), were in part supported by the cluster analysis (HCA). The HCA was based on a different defined distance between sets (the Euclidean distance). The obtained dendrogram of cultivars demonstrated that all cultivars could be clustered into five groups (Figure 6). In the biggest group, RED, three different subsets could be distinguished. All of them were only composed of LA cultivars. The common feature connecting all cultivars in the RED group was the similar level of linalool (29.2–44.1%) and camphor (0.19–0.77%). The subsets in the RED group were differentiated by the content of cymene (0.11–0.34%), geranyl acetate (0.77–1.81%) (subset LA2/TF to A2/2018), *β*-myrcene (0.36–0.51%) (subset LA5 to A12), and terpinen-4-ol (10.6–11.2%) (subset LA6 and A3). The YELLOW group only consisted of lavandin cultivars, except for cultivar LI2 that was very distinct from all other lavender cultivars and was not clustered (analogically to PCA results). The PURPLE group contained four cultivars of true lavender distinguished by a high content of *cis*-linalool oxide (0.5–1.8%) and matching levels of the sum of butyl butanoate (0.28–0.51%) and α-terpineol (2.4–4.5%). The remaining two groups, black and blue, consisted of two cultivars each, for which the content of linalool was similar.

**Figure 7:**
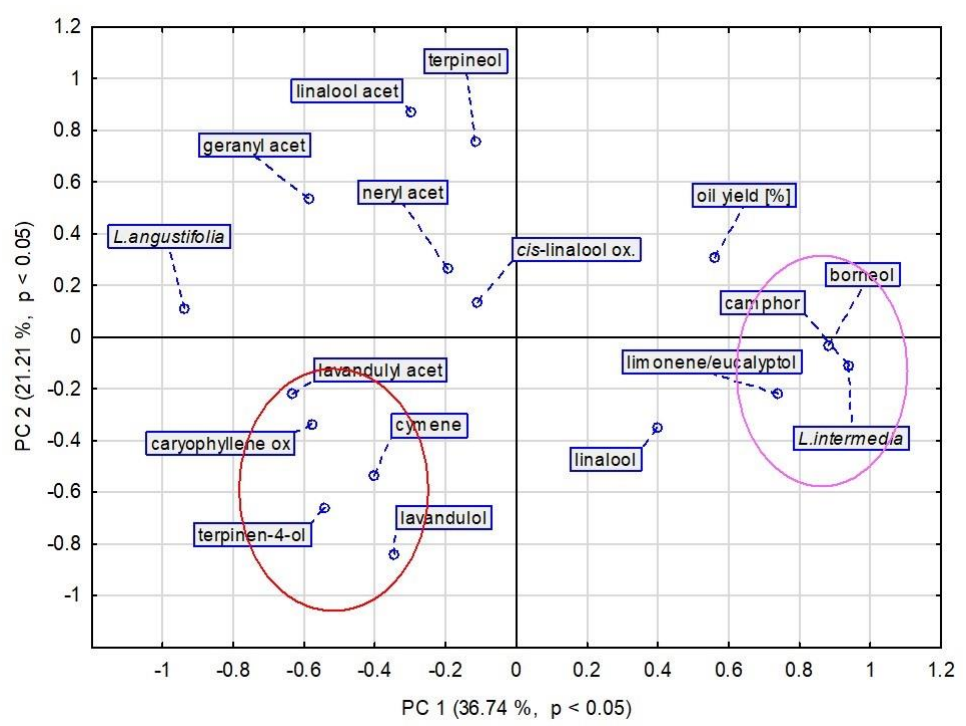
PCA score plot of all obtained data based on average content of EO components in different species. PC—principal component.

**Figure 8:**
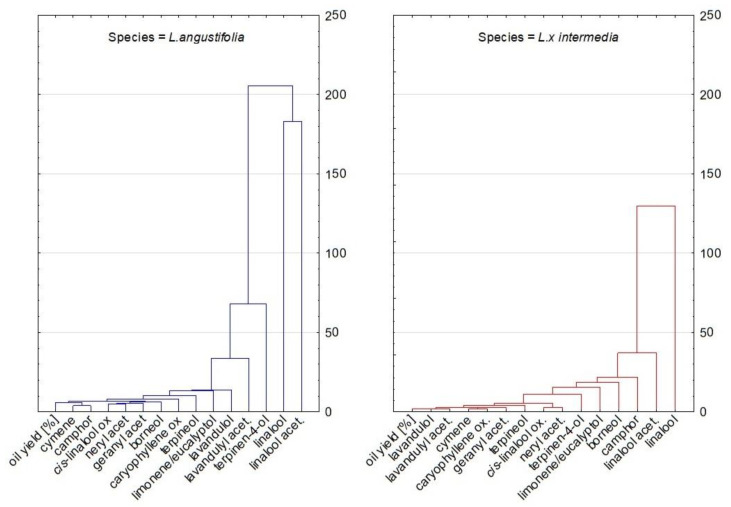
Dendrograms obtained via the HCA analysis of all obtained data based on average content of EO components in species.

Further statistical analyses were focused on interspecific differences. Figure 7 presents a PCA plot with a differential analysis of EO components between species, and Figure 8 shows analogic HCA dendrograms. These figures confirm our previous observations. For *L. x intermedia* essential oils, the differentiating compounds with a higher content than in *L. angustifolia* were found to be camphor, borneol, and the sum of limonene and eucalyptol (in fact eucalyptol). For *L. angustifolia*, higher levels of terpinen-4-ol, lavandulyl acetate, lavandulol, caryophyllene oxide, and cymene were significant for differentiation from LI. These conclusions were additionally confirmed by a multivariate parametric analysis of variance (MANOVA). All the interspecies differences of the above-mentioned compounds were statistically significant (*p* < 0.0001). Higher camphor, borneol, and eucalyptol contents in lavandin oil are well known. The same refers to the higher yield of lavandin oils. These results were in line with the literature. The mentioned components and oil yield are discussed in detail in Part A of this paper.

We can additionally observe that the two main components, linalool and linalyl acetate, were very distant from each other on dendrograms and the PCA plot. They are opposed to each other, as shown by the way linalool transforms into linalyl acetate in the biosynthesis pathway and vice versa, especially during the prolonged steam oil distillation [31,33].

The above-described statistical analyses confirmed the fact that the differentiating compounds for *L. x intermedia* are camphor, borneol, and eucalyptol. In addition, the analyses highlighted caryophyllene oxide as a potential differentiating compound for the studied species. This is very interesting discovery since, according to our knowledge, this compound has never been indicated in the literature as a true lavender/lavandin oil differentiator. As a minor component, it is most often skipped in analyses. Reviewing available data, we can observe that caryophyllene oxide was detected in LA oils at levels of up to 8.7%, with the most common values of around 2% [11]. For *L. x intermedia*, there is less available data. Detar et al. analyzed the LA and LI essential oil compositions of several cultivars grown in Hungary and reported ranges 0.2–3.3% and 0–1.4%, respectively [15]. Kara et al. reported caryophyllene oxide in lavandin oil in the range of 0–0.85%, depending on the distillation time and fraction [21]. Popa et al. reported 0.4% and 0.1% of caryophyllene oxide in true lavender and lavandin oil, respectively [34]. The reported literature data support the hypothesis that caryophyllene oxide could be another true lavender/lavandin oil-differentiating marker. However, more studies are needed to prove this hypothesis, especially for oils originating from different cultivars and cultivation places.

## 3. Materials and Methods

### 3.1. Plant Material

Flowering parts of *Lavandula angustifolia* were collected from experimental plots at the “Novokakhovske” farm in 2016, 2017, 2018, and 2020. The voucher specimens of each year were deposited at the Herbarium of the Sector of Mobilization and Conservation of Plant Resources of the Rice Institute of the NAAS (Plodove, Kherson region). The information about the cultivars studied is provided in Table 8. All the oil yields presented in the table were calculated for a fresh mass.

### 3.2. Hydrodistillation

The essential oils from fresh flowers of *Lavandula angustifolia* were produced in a Clevenger-type apparatus via hydrodistillation for 4 hours. The oils were kept at room temperature in sealed tubes (light-protected) before being subjected to analysis with GC–MS.

### 3.3. GC–MS Analysis of EOs

EOs were analyzed with the same method and instrument as in our previous work [18]. We used a Hewlett Packard gas chromatograph (HP 6890 series GC) with a Hewlett Packard 5973 mass detector. The instrument was equipped with a high-temperature ZB-5HT (5% diphenyl- and 95% dimethyl-polysiloxane) 30 m long capillary column with an inner diameter of 0.32 mm and a film thickness of 0.25 μm (Phenomenex Inc., Torrance, CA, USA). EOs (20 µL) were diluted with dichloromethane (Sigma-Aldrich ACS Grade, Merck, Darmstadt, Germany) and directly analyzed. We injected 1 μL of a sample using the a split injection technique with a split ratio of 20:1 and an injector temperature of 250 °C. Helium was used as the carrier gas, and its flow rate was 2 mL/min. The temperature oven program was 40–180 °C with a heating rate of 5 °C/min and 180–280 °C with a heating rate of 10 °C/min. The solvent delay was three minutes. Peaks were identified by comparing the mass spectra data with the NIST 11 Library and by comparing their retention indices with literature values. In some cases, comparisons with standards were necessary. Retention index mixture, the terpineol mixture of isomers, and sabinene hydrate analytical standards were obtained from Merck. Amounts of the detected components represent % abundance (area percent, solvent peak excluded).

### 3.4. Statistical Analysis

All the analyses were carried out in triplicate, and the results were expressed as means. Due to the large set of data, standard deviations are only presented in a large supplementary table (Table S1). The statistical analyses were performed using STATISTICA 13.1 software (StatSoft Inc., Tulsa, OK, USA). The obtained data were analyzed using hierarchical cluster analysis (full linkage using Euclidean distance) and principal component analysis (PCA). The PCA model (based on standardized data) was estimated using the NIPALS iterative algorithm. The criterion of convergence was set at the level of 0.00001, and the maximum number of iterations was set at 50. The number of components was determined by determining the maximum predictive capability using the method of multiple cross-validation, and the maximum number of components was set at the level. The obtained optimal PCA model was then reduced to 2 components. The conducted PCA, the results of which are presented on the chart of PC 1 vs. PC2 loads, allowed us to select variables with the most significant influence on the variability of the analyzed database of results and to select the most significant correlations between them. The variables selected in this way were then subjected to further statistical evaluation. These two classification techniques (PCA and HCA) were used to discover natural groupings in the data and examine differences between the analyzed lavender species. A parametric multivariate analysis of variance (ANOVA) was used to compare compound content between years and to compare variation in LA and LI species across years. All variables met the assumption of normality of distribution and homogeneity of variance. The normality of distribution of the compared variables was tested by three different statistical tests: the Kolmogorov–Smirnov test, the Lilliefors test, and the W Shapiro–Wilk test. The homogeneity of variance was assessed using the Brown–Forsythe and Levene’s tests. Statistical significance was considered when *p* < 0.05. Comparisons of LA and LI cultivars in terms of area abundance (%) were assessed with *t* tests. Correlations between all cultivars were tested using the Spearman rank-order correlation method.

## 4. Conclusions

The chemical composition of seven new cultivars of *L. angustifolia* and eight new cultivars of *L. x intermedia* was investigated. Over seventy different components were identified or tentatively identified, and they are presented in the extensive tables. Linalool and linalool acetate were the main oil constituents for both species. Notably, the *L. angustifolia* cultivars yielded oils with a high linalool content, some in the maximum of the range reported in the literature. Another interesting feature of the studied true lavender oils was the high content of terpinen-4-ol. This is in line with our previous results and appears to be a characteristic of *L. angustifolia* oils bred in Ukraine. Despite valuable terpene profiles, the new cultivars produce oils that do not comply with industry standards. If the cultivation of lavender in Ukraine is still growing, it will be beneficial to consider adding a “Ukrainian origin” chromatographic profile specification to the current industry standard. Regarding the studied cultivars of *L. x intermedia,* the two main components were accompanied by a significant presence of camphor, borneol, and (in some cases) eucalyptol. To the best of our knowledge, this is the first research work that characterizes the composition of lavandin oil of Ukrainian origin. The comparative analysis of the oils from both species grown on the same plots revealed that lavandin oils contained much higher contents of camphor, borneol, and eucalyptol than true lavender oils. These results are in line with other findings and are typical differences between lavender and its hybrids. Furthermore, the conducted statistical analyses marked caryophyllene oxide as an another potential differentiating compound for the studied species. This hypothesis must be verified on oils of other origin. All the gathered results enable us to characterize the developed cultivars and to select ones with not only the best morphological/physiological features, crops, and oil yields but also chemical characteristics.

## Figures and Tables

**Figure 3:**
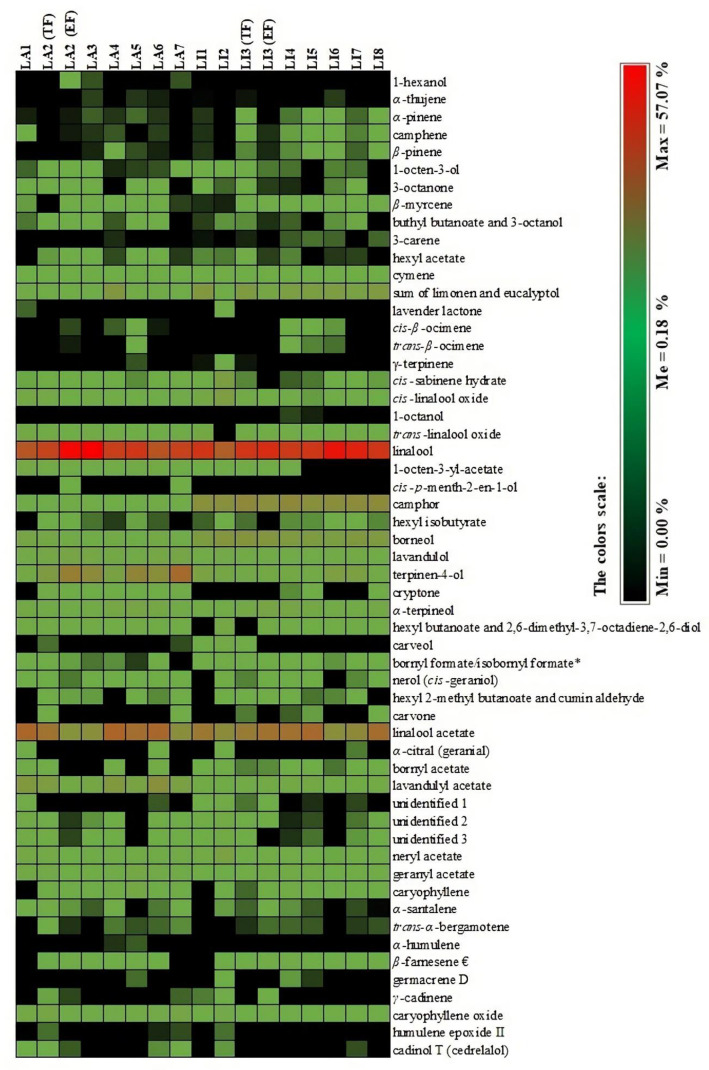
A heatmap of tested LA and LI essential oil samples based on the compounds identified with GC–MS. Black color indicates that the compound was not detected in the sample. * tentative identification.

**Table 1:** Normative content requirements (in %) for *L. angustifolia*, *L. latifolia*, and *L. x intermedia* essential oil chromatographic profiles.

Component	*L. angustifolia*		*L. latifolia*	*L. x intermedia*	
	No Origin Distinction	Other Origins	Spanish Type	‘Abrial’	‘Grosso’
	Ph. Eur.	ISO 3513	ISO 4719	ISO 3054	ISO 8902
linalool	20–45	20–43	34–50	28–38	24–37
linalyl acetate	25–47	25–47	0–1.6	19–29	25–38
camphor	0–1.2	0–1.5	8–16	7–11	6.5–8
borneol	–	–	0.5–3	1.5–3.5	1.5–3.5
limonene	0–1	0–1	0.5–3	0.5–1.5	0.5–1.5
eucalyptol	0–2.5	0–3	16–39	6–12.5	4–8
*Β*-phellandrene	–	0–1	–	–	–
terpinen-4-ol	0.1–8	0–8	–	0.3–1.2	1.5–5
*α*-terpineol	0–2	0–2	0.2–2	0.3–1.2	0.3–1.3
lavandulyl acetate	min. 0.2	0–8	–	1–2	1.5–3.5
lavandulol	min. 0.1	0–3	–	0.4–1.2	0.2–1
hexyl butyrate	–	–	–	0.2–0.5	0.3–0.5
caryophyllene	–	–	–	1.5–2.5	–
*β*-myrcene	–	–	–	0.4–0.9	0.3–1
3-octanone	0.1–5	0–3	–	–	–
*cis-β*-ocimene	–	1–10	–	1.4–3	0.5–1.5
*trans-β*-ocimene	–	0.5–6	–	2.5–6	0–1
*trans-α*-bisabolene	–	–	0.4–2.5	–	–

Numeric values in %; min.—minimum.

**Table 2:** Relative abundances (in %) of components in essential oils from seven new different *Lavandula angustifolia* cultivars (LA1-LA7) cultivated in 2020. Reference LRI (linear retention index) values were sourced from [24,25]; * tentative identification, TF—total flowering, exp.—experimental, ref.—reference, nd—no data.

Component		LRI		Cultivar							
				LA1	LA2		LA3	LA4	LA5	LA6	LA7
		LRI exp.	LRI ref.	TF	TF	End of TF	TF	TF	TF	TF	TF
**1**	1-hexanol	869	870			0.18	0.07				0.07
2	*α*-thujene	924	928				0.06		0.05	0.03	
3	*α*-pinene	929	936	0.03		0.02	0.09	0.05	0.01	0.05	
4	camphene	942	950	0.19		0.02	0.05	0.08		0.06	
5	*β*-pinene	970	978				0.05	0.23	0.07	0.03	
7	1-octen-3-ol	981	980	0.09	0.42	0.36	0.27	0.04	0.06	0.07	0.40
8	3-octanone	986	985	0.15	0.40	0.50	0.36		0.23	0.31	
9	*β*-myrcene	991	989	0.14		0.20	0.19	0.18	0.38	0.22	0.06
10	butyl butanoate	997	997					0.08	0.34	0.27	
11	3-octanol	998	993	0.11	0.20	0.34					
12	coelution of butyl butanoate and 3-octanol	998					0.22				
13	3-carene	1007	1011					0.04			
14	hexyl acetate	1015	1010		0.14	0.22	0.17	0.07	0.34	0.26	0.05
15	p-cymene	1022	1024	0.17	0.23	0.38	0.43	0.19	0.44	0.41	0.19
16	coelution of eucalyptol and limonene	1026		1.51	0.72	0.81	0.53	7.79	0.57	1.07	0.38
17	lavender lactone	1039	1039	0.09							
18	*cis-β*-ocimene	1041	1038			0.06		0.08	0.82	0.02	
19	*trans-β*-ocimene	1047	1048			0.03			0.42		
20	*γ*-terpinene	1056	1060						0.07		
21	*cis*-sabinene hydrate	1065	1065	0.15	0.27	0.29	0.32	0.24	0.12	0.24	0.61
22	*cis*-linalool oxide	1070	1075	2.32	1.36	0.43	1.15	0.71	0.25	0.49	0.91
23	1-octanol	1075	1072								
24	*trans*-linalool oxide	1086	1083	2.09	1.31	0.55	1.12	0.67	0.25	0.50	0.89
25	linalool	1105	1099	29.51	35.38	55.08	57.07	36.60	39.50	29.87	35.98
26	1-octen-3-yl-acetate	1114	1110	1.79	0.76	0.42	0.51	0.35	0.82	1.24	0.35
27	*cis-* p-menth-2-en-1-ol	1120	1123			0.40					0.26
28	camphor	1139	1141	0.90	0.54	0.95	0.40	0.45	0.32	0.97	0.32
29	hexyl isobutyrate	1149	1150		0.18	0.15	0.10	0.06	0.14	0.08	
30	borneol	1163	1166	3.16	2.41	1.51	0.74	1.40	0.53	1.76	0.80
31	lavandulol	1168	1168	1.37	2.44	3.14	2.34	2.05	1.30	2.35	2.62
32	terpinen-4-ol	1175	1177	2.16	6.52	15.80	11.73	4.15	13.76	10.65	22.44
33	cryptone	1183	1184		0.97	0.47	0.32	0.36	0.33	0.34	0.65
34	p-cymen-8-ol	1183	1184	0.08							
35	*α*-terpineol	1189	1192	1.85	1.20	0.94	1.19	2.16	3.37	2.21	1.06
36	hexyl butanoate	1192	1196			0.83	0.97	0.76	0.98	0.84	0.57
37	coelution of hexyl butanoate and 2,6-dimethyl-3,7-octadiene- 2,6-diol	1193			1.27						
38	2,6-dimethyl-3,7-octadiene-2,6-diol	1196	1190	1.14							0.22
39	carveol	1219	1219		0.10						0.07
40	bornyl formate/isobornyl formate *	1223	1229	0.30	0.23	0.14	0.11	0.13	0.05	0.18	
41	nerol (*cis*-geraniol)	1229	1236	0.27	0.24	0.11	0.16	0.20	0.47	0.31	0.21
42	hexyl 2-methyl butanoate	1237	1234				0.14				
43	coelution of hexyl 2-methyl butanoate and cumin aldehyde	1237			0.43	0.14			0.23	0.12	
44	cumin aldehyde	1237	1239								0.17
45	neral (=*β*-citral)	1240	1242	0.23							
46	carvone	1241	1242		0.30						0.18
48	unidentified	1249		0.24	0.76	0.51	0.64	0.23	0.10	0.60	1.91
50	linalyl acetate	1257	1255	23.81	19.43	9.08	10.51	24.45	20.89	23.27	10.87
51	*α*-citral (geranial)	1270	1270	0.33						0.32	
52	2,6-dimethylocta-1,7-diene-3,6-diol	1279	1286	0.34							
53	bornyl acetate	1282	1284	0.87	0.47			0.70		0.70	
54	lavandulyl acetate	1291	1289	7.38	5.91	2.12	2.65	7.19	3.79	10.23	4.58
55	unidentified	1344		0.76						0.08	
56	unidentified	1352		1.18	0.51	0.05	0.13	0.25		0.24	0.24
57	unidentified	1354		1.20	0.59	0.06	0.16	0.34		0.31	0.27
58	neryl acetate	1364	1363	2.42	1.38	0.26	0.83	0.71	1.09	0.93	1.00
59	geranyl acetate	1384	1380	2.05	1.65	0.47	0.70	0.93	2.15	1.54	0.87
60	unidentified	1393		0.24						0.35	0.78
61	caryophyllene	1412	1420		0.23	0.49	0.19	0.65	2.15	0.51	0.39
62	*α*-santalene	1414	1421	0.30	0.60	0.13	0.08	0.36		0.11	0.28
63	*trans-α*-bergamotene	1431	1434		0.19	0.05		0.10	0.07	0.09	0.12
64	*α*-humulene	1446	1453					0.04	0.08		
65	*trans-β*-farnesene	1455	1456		0.52	0.65	0.28	0.16	0.32	0.20	
66	germacrene D	1473	1481						0.10		
67	unidentified			1.45	0.51				0.04		0.29
68	*γ*-cadinene	1508	1513		0.14	0.06					0.09
69	caryophyllene oxide	1574	1581	2.00	2.92	0.93	0.85	1.73	1.15	1.51	2.59
70	humulene epoxide II	1601	1606		0.10					0.03	0.07
71	cadinol T (cedrelalol)	1637	1635	0.24	0.49	0.08				0.13	0.20
72	*α*-cadinol *	1668	1659		0.08						0.06
73	*α*-bisabolol	1682	1683							0.16	
74	geranyl-p-cymene *	1955	nd		0.06						

**Table 3:** The top 10 abundant components in essential oils of the studied *L. angustifolia* cultivars. The compounds are ordered according to the total sum of % area for all the cultivars. Numbers are relatives abundancies in %.

Rank	Component	Cultivar							
		LA1	LA2		LA3	LA4	LA5	LA6	LA7
		TF	TF	End of TF	TF	TF	TF	TF	TF
1	linalool	29.51	35.38	55.08	57.07	36.60	39.50	29.87	35.98
2	linalyl acetate	23.81	19.43	9.08	10.51	24.45	20.89	23.27	10.87
3	terpinen-4-ol	2.16	6.52	15.80	11.73	4.15	13.76	10.65	22.44
4	lavandulyl acetate	7.38	5.91	2.12	2.65	7.19	3.79	10.23	4.58
5	lavandulol	1.37	2.44	3.14	2.34	2.05	1.30	2.35	2.62
6	*α*-terpineol	1.85	1.20	0.94	1.19	2.16	3.37	2.21	1.06
7	caryophyllene oxide	2.00	2.92	0.93	0.85	1.73	1.15	1.51	2.59
8	eucalyptol + limonene (small)	1.51	0.72	0.81	0.53	7.79	0.57	1.07	0.38
9	borneol	3.16	2.41	1.51	0.74	1.40	0.53	1.76	0.80
10	geranyl acetate	2.05	1.65	0.47	0.70	0.93	2.15	1.54	0.87

**Table 4:** Relative abundances (in %) of components in the essential oils obtained in eight new *Lavandula x intermedia* cultivars (LI1-LI8). * tentative identification.

Component		LRI		Cultivar								
				LI1	LI2	LI3		LI4	LI5	LI6	LI7	LI8
		LRI exp.	LRI ref.	2016	2016	2017	2018	2020	2020	2020	2020	2020
2	*α*-thujene	924	928	0.01		0.02				0.06		
3	*α*-pinene	929	936	0.05		0.18		0.11	0.22	0.35	0.09	0.20
4	camphene	942	950	0.04		0.23	0.04	0.14	0.30	0.26	0.11	0.26
5	*β*-pinene	970	978	0.05		0.12	0.04	0.12	0.19	0.34	0.09	0.23
6	Sabinene *	975	973	0.04		0.03						
7	1-octen-3-ol	981	980	0.24	0.21	0.35	0.11	0.10		0.11	0.10	
8	3-octanone	986	985	0.16	0.09	0.28	0.06	0.04		0.12	0.18	
9	*β*-myrcene	991	989	0.04	0.03	0.16	0.20	0.30	0.27	0.29	0.18	0.16
10	butyl butanoate	997	997	0.06		0.13	0.04	0.09		0.13		
11	3-octanol	998	993		0.13							
12	coelution of butyl butanoate and 3-octanol	998									0.14	
13	3-carene	1007	1011	0.02		0.03		0.08	0.10	0.09		0.09
14	hexyl acetate	1015	1010	0.12	0.11	0.25	0.06	0.12		0.05	0.06	
15	p-cymene	1022	1024	0.26	0.59	0.29	0.17	0.19	0.20	0.26	0.19	0.17
16	coelution of eucalyptol and limonene	1026		7.41	0.47	6.24	2.65	5.00	5.09	6.01	3.98	6.71
17	lavender lactone	1039	1039		0.34							
18	*cis-β*-ocimene	1041	1038					0.35	0.23	0.13		
19	*trans-β*-ocimene	1047	1048					0.44	0.12	0.10		
20	*γ*-terpinene	1056	1060	0.02	0.33	0.02						
21	*cis*-sabinene hydrate	1065	1065	0.33	5.66	0.12		0.08	0.11	0.20	0.18	0.14
22	*cis*-linalool oxide	1070	1075	2.21	5.35	1.20	0.67	0.20	0.14	0.21	0.54	0.42
23	1-octanol	1075	1072					0.06	0.03			
24	*trans*-linalool oxide	1086	1083	2.07		1.14	0.72	0.27	0.21	0.28	0.67	0.51
25	linalool	1105	1099	40.16	26.14	39.85	42.93	38.38	39.15	51.24	46.21	40.17
26	1-octen-3-yl-acetate	1114	1110	0.32	0.26	0.44	0.44	0.51				
28	camphor	1139	1141	10.11	10.13	12.55	10.21	12.27	10.32	10.61	11.32	11.91
29	hexyl isobutyrate	1149	1150	0.09	0.27	0.10		0.12	0.13	0.16	0.15	0.12
30	borneol	1163	1166	6.31	8.69	8.71	7.86	6.63	6.20	5.49	7.33	6.42
31	lavandulol	1168	1168	0.60	1.57	0.98	0.93	0.89	0.65	1.26	1.43	0.59
32	erpinene-4-ol	1175	1177	4.37	2.65	2.12	1.85	1.86	1.80	5.35	4.94	1.54
33	cryptone	1183	1184					0.12	0.16			0.18
35	*α*-terpineol	1189	1192	1.14	0.58	2.00	3.42	2.73	1.98	1.15	1.25	1.83
36	hexyl butanoate	1192	1196					0.70	0.74	0.63	0.91	0.72
38	2,6-dimethyl-3,7-octadiene-2,6-diol	1196	1190		2.24		0.91					
39	carveol	1219	1219	0.91	1.35	0.92						
40	bornyl formate/isobornyl formate *	1223	1229	0.29	0.55	0.17	0.18	0.17	0.18	0.13	0.21	0.18
41	nerol (*cis*-geraniol)	1229	1236		0.69	0.12	0.28	0.24	0.25		0.12	0.16
42	hexyl 2-methyl butanoate	1237	1234		0.25	0.17	0.19			0.12	0.20	
43	hexyl 2-methyl butanoate and cumin aldehyde coelution	1237						0.15	0.10			
45	neral (=*β*-citral)	1240	1242				0.11					
46	carvone	1241	1242			0.11		0.08	0.14			0.15
47	hexyl 3-methyl butanoate(=n-Hexyl iso-valerate)/n-valerate *	1242	1244							0.09	0.13	
48	unidentified	1249				0.12		0.02	0.05	0.09	0.23	0.07
49	thymoquinone	1255	1260				0.23					
50	linalyl acetate	1257	1255	17.55	11.51	16.50	19.67	19.86	22.47	10.57	12.10	21.23
51	*α*-citral (geranial)	1270	1270		0.22						0.11	
52	2,6-dimethylocta-1,7-diene-3,6-diol	1279	1286		0.88							
53	bornyl acetate	1282	1284	0.18	0.28	0.11	0.12	0.20	0.37	0.10	0.21	0.40
54	lavandulyl acetate	1291	1289	1.12	1.14	1.15	1.47	1.80	1.81	1.15	1.31	1.44
55	unidentified	1344		0.22	1.21	0.10	0.15		0.04		0.06	
56	unidentified	1352		0.41	1.36	0.20	0.18	0.04	0.07		0.11	0.15
57	unidentified	1354		0.40	1.17	0.22		0.05	0.10		0.13	0.20
58	neryl acetate	1364	1363	1.43	4.25	0.80	0.72	0.57	0.60	0.15	0.59	0.51
59	geranyl acetate	1384	1380	0.47	0.54	0.57	1.33	0.98	1.10	0.25	0.75	0.71
60	unidentified	1393			0.59							
61	caryophyllene	1412	1420		0.52	0.09	0.21	0.55	0.60	0.34	0.25	0.30
62	*α*-santalene	1414	1421		0.14	0.09	0.19	0.13	0.08		0.07	0.01
63	*trans-α*-bergamotene	1431	1434			0.05	0.10	0.09	0.08		0.05	0.07
64	*α*-humulene	1446	1453									
65	*trans-β*-farnesene	1455	1456		0.20	0.16	0.70	0.76	0.24	0.26	0.19	0.19
66	germacrene D	1473	1481		0.28			0.14	0.05			
67	unidentified			0.44	1.54	0.16						
68	*γ*-cadinene	1508	1513	0.11	0.51		0.26					
69	caryophyllene oxide	1574	1581	0.24	1.52	0.36	0.61	0.34	0.33	0.21	0.62	0.48
70	humulene epoxide II	1601	1606		0.10							
71	cadinol T (cedrelalol)	1637	1635								0.06	
73	*α*-bisabolol	1682	1683						0.45		0.40	0.42

**Table 6:** Comparative table for the chemical composition of EOs obtained from new Ukrainian cultivar of *L. x intermedia,* with the normative content values given different ISO norms for *L. x* *intermedia* and its parent species *L. angustifolia* and *L. latifolia*.

Component	Area abundance [%]			Normative Range [%]				Comparison to ISO Normative Values
	*L. x intermedia*			*L. x intermedia*		*L. angustifolia*	*L. latifolia*	
	Ukrainian Origin			‘Abrial’	‘Grosso’	Other Origin	Spanish Type	
	Max	Min	Mean	ISO 3054	ISO 8902	ISO 3513	ISO 4719	
linalool	51.2	26.1	40.5	28–38	24–37	20–43	34–50	Generally higher than ‘Abrial’, ‘Grosso’, slightly higher than LA, similar to LL (*L. latifolia*)
linalyl acetate	22.5	10.6	16.8	19–29	25–38	25–47	0–1.6	Much lower than ‘Grosso’ and LA, lower than ‘Abrial’, much higher than LL
camphor	12.6	10.1	11.0	7–11	6.5–8	0–1.5	8–16	Higher than ‘Grosso’, little higher than ‘Abrial’, similar to spike; much higher than LA
borneol	8.7	5.5	7.1	1.5–3.5	1.5–3.5	nr	0.5–3	Much higher than LL, ‘Abrial’ and ‘Grosso’
limonene	c	c	c	0.5–1.5	0.5–1.5	0–1	0.5–3	Small limonene content or traces, unable to quantify due to coelution
eucalyptol	c	c	c	6–12.5	4–8	0–3	16–39	Eucalyptol was dominant in this coelution
eucalyptol and limonene *	7.4	0.5	4.8	6.5–14	4–8	0–4	16.5–42	Variable and similar to ‘Grosso’, much less than spike and less than ‘Abrial’; higher than LA
terpinen-4-ol	5.3	1.5	2.9	0.3–1.2	1.5–5	0–8	nr	Lower limit of LA, higher than LL and ‘Abrial’, similar to ‘Grosso’
*α*-terpineol	3.4	0.6	1.8	0.3–1.2	0.3–1.3	0–2	0.2–2	Upper limit of LA, higher than ‘Abrial’ and ‘Grosso’
lavandulyl acetate	1.8	1.1	1.4	1–2	1.5–3.5	0–8	nr	Generally in all referenced ISO limits, similar to ‘Abrial’
lavandulol	1.6	0.6	1.0	0.4–1.2	0.2–1	0–3	nr	Generally in all referenced ISO limits, similar to ‘Abrial’ and ‘Grosso’
hexyl butanoate and 2,6-dimethyl-3,7-octadiene-2,6-diol **	2.2	0.6	1.0	0.2–0.5	0.3–0.5	nr	nr	Close to ‘Abrial’ and ‘Grosso’
caryophyllene	0.6	0.1	0.4	1.5–2.5	nr	nr	nr	Lower than ‘Abrial’
*β*-myrcene	0.3	0,0	0.2	0.4–0.9	0.3–1	nr	nr	Lower than ‘Abrial’ and ‘Grosso’
3-octanone	0.3	0.0	0.1	nr	nr	0–3	nr	In the lower range of LA specification
*cis-β*-ocimene	0.3	0.1	0.2	1.4–3	0.5–1.5	1–10	nr	Lower than LA, ‘Grosso’ and ‘Abrial’
*trans-β*-ocimene	0.4	0.1	0.2	2.5–6	0–1	0.5–6	nr	Lower than LA, ‘Abrial’, similar to ‘Grosso’
*trans-α*-bisabolene	un	un	un	nr	nr	nr	0.4–2.5	Lower than LL

* Due to a coelution of limonene and eucalyptol, they are presented as a sum. *β*-phellandrene was not detected in the studied samples, but its traces might have coeluted with limonene and eucalyptol. ** Due to a coelution of hexyl butanoate and 2,6-dimethyl-3,7-octadiene-2,6-diol, they are presented as a sum. Un—undetected, nr—not regulated, c—coelution, LA—*L. angustifolia*, LL—*L. latifolia* (spike).

**Table 7:** Comparative table of essential oils from the studied new Ukrainian cultivars of lavandin with the new Ukrainian cultivars of *L. angustifolia* grown on the same plots. The table includes regulated components and/or some main component identified in tested samples. The marked *p* values (in bold) are statistically significant.

Component	Area Abundance [%]						Comparison to Studied LA		*T*-test *p*-Value
	*L. x intermedia* (LI)			*L. angustifolia* (LA)					
	Max	Min	Mean	Max	Min	Mean			
camphor	12.6	10.1	11.0	1.0	0.3	0.6	higher	**↑↑**	**<0.0001**
borneol	8.7	5.5	7.1	3.2	0.5	1.5	higher	**↑↑**	**<0.0001**
eucalyptol and limonene	7.4	0.5	4.8	7.8	0.4	1.7	higher	**↑**	**<0.0001**
carveol	1.4	0.9	1.1	0.1	0.1	0.1	higher	**↑**	**<0.0001**
linalool	51.2	26.1	40.5	57.1	29.5	39.9	similar	≈	0.8066
linalyl acetate	22.5	10.6	16.8	24.4	9.1	17.8	similar	≈	0.5198
*α*-terpineol	3.4	0.6	1.8	3.4	0.9	1.7	similar	≈	0.8589
*cis*-linalool oxide	5.3	0.1	1.2	2.3	0.3	1	similar	≈	0.0912
neryl acetate	4.3	0.1	1.1	2.4	0.3	1.1	similar	≈	0.1389
hexyl butanoate and 2,6-dimethyl-3,7-octadiene-2,6-diol	2.2	0.6	1.0	1.3	0.8	0.9	similar	≈	0.7679
trans-linalool oxide	2.1	0.2	0.7	2.1	0.3	0.9	similar	≈	0.2586
*β*-myrcene	0.3	0	0.2	0.4	0.1	0.2	similar	≈	0.6951
*cis-β*-ocimene	0.3	0.1	0.2	0.8	0	0.2	similar	≈	0.9160
*trans-β*-ocimene	0.4	0.1	0.2	0.4	0	0.2	similar	≈	0.9655
terpinen-4-ol	5.3	1.5	2.9	22.4	2.2	10.9	lower	**↓**	**<0.0001**
lavandulyl acetate	1.8	1.1	1.4	10.2	2.1	5.5	lower	**↓**	**<0.0001**
lavandulol	1.6	0.6	1.0	3.1	1.3	2.2	lower	**↓**	**<0.0001**
geranyl acetate	1.3	0.3	0.7	2.1	0.5	1.3	lower	**↓**	**0.0001**
caryophyllene oxide	1.5	0.2	0.5	2.9	0.9	1.7	lower	**↓**	**<0.0001**
caryophyllene	0.6	0.1	0.4	2.2	0.2	0.7	lower	**↓**	**0.0336**
3-octanone	0.3	0	0.1	0.5	0.2	0.3	lower	**↓**	**<0.0001**

**Table 8:** Information about the studied cultivars of *L. angustifolia* and *L. x intermedia*.

No	Cultivar Name	Sample ID	Characteristic	Vegetation Phase of Plant	Date of Oil Production	Oil Yield [%]
*L. angustifolia*						
**LA1**	Record	14/460	The cultivar was created in the Nikitsky Botanical Garden. Plants are large: 55–60 cm tall and 60–65 cm in diameter. The shape of shrubs is semi-spreading. Peduncles are straight, thin, elastic, green, and 20–22 cm long. Inflorescences are cylindrical with 6–7 rings that are 7-8 cm long. Corollas are purple, and calyxes are purple–green. The mass fraction of essential oil varies from 1.2% to 1.5% depending on the weather conditions of the year.	Total flowering	06 July 2020	1.25
**LA2**	Sineva Nadii	15/452	Shrubs of a medium size, with a compact shape, 60 cm height, and 80 cm in diameter. Flowering shoots are straight and green. Their thickness is average. Inflorescences are elongated and 12–14 cm long. Calyxes are purple. Corollas are deep purple and moderately pubescent. Inflorescences have diameters of 2 cm. The number of rings is 10–11. The leaves are narrow gray–green, 5.0–5.5 cm long, and 0.4–0.5 cm wide. The mass fraction of essential oil ranges from 0.95% to 1.3% of the fresh mass. It is included in the Register of Cultivars of Ukraine and the base of the data of the genofund information system of plants in the National Center of Genetic Resources of Plants of Ukraine.	Total flowering	24 June 2020	0.50
		15/473		The end of total flowering	10 July 2020	0.96
**LA3**	№ 463-20	17/463	Shrubs are large, semi-spreading, 80 cm tall, and 90 cm in diameter. Flowering shoots are straight, green, and 30 cm long. Their thickness is average. Inflorescences are 6 cm long. Calyxes are green. Corollas are blue. The diameter of inflorescence is 2 cm. The number of rings is 7. Leaves are narrow gray–green, 5.0–5.5 cm long, and 0.5–0.7 cm wide. The mass fraction of essential oil varies from 1.0% to 1.25% of the fresh mass.	Total flowering	07 July 2020	1.25
**LA4**	Zmijuchka	18/464	Bushes have a spherical shape, feature spreading, and differ from other plants in their extremely thin and wavy peduncles. The height and diameter of bushes are 55–60 and 80–90 cm, respectively. Leaves are 3.0–3.5 cm long, 0.4–0.5 cm wide, and green with a gray tinge. Inflorescences are light blue, 6.0–7.0 cm long, and 1.5–1.8 cm in diameter. The essential oil content is about 1.3% of the fresh mass.	Total flowering	08 July 2020	1.10
**LA5**	№ 1-469	19/469	Shrubs are 60–65 cm tall and 60–70 cm in diameter. Flowering shoots are straight, green, and 20–25 cm long. Their thickness is average. Inflorescences are 6 cm long. The number of rings is 6–7. The calyx is purple–green. The corolla is purple. The mass fraction of essential oil varies from 0.7% to 0.9% of the fresh mass.	Total flowering	09 July 2020	0.70
**LA6**	№ 1-2-20	20/471	Shrubs are of medium size and compact shape. Their height is 50–60 cm and 70–80 cm in diameter. Flowering shoots are straight, green, and 20–25 cm in length. Their thickness is medium. Inflorescences are cylindrical. Their length is 5–6 cm. The corolla is purple, and the calyx is purple–green. The mass fraction of essential oil ranges from 0.5% to 0.7% of the fresh mass.	Total flowering	09 July 2020	0.60
**LA7**	Pink flamingo	21/458	Bushes are 65 cm high and 105 cm in diameter. Leaves are 5.7–6.0 cm long and 0.6–0.7 cm wide. Inflorescences are 6.0–6.5 cm long and 2.0 cm in diameter. Corollas are pink. The content of essential oil is 0.7–0.8% of the fresh mass; it is included in the base of the data of the genofund information system of plants in the National Center of Genetic Resources of Plants of Ukraine. This cultivar can be used for landscaping. It differs from *Lavandula angustifolia* Rosea in terms of the habit of bushes.	Total flowering	03 July 2020	0.70
***L. x intermedia***						
**LI1**	Etjod	1/130	Shrubs are large, compact in shape, 100 cm high, and 90–100 cm wide. Inflorescences are cylindrical, dense, and 9–11 cm long with 7 rings. Corollas are blue and moderately pubescent. The average ring has from 10 to 25 flowers (16 flowers on average). Leaves are linear gray–green, slightly pubescent, 4.5–6.0 cm long, and 0.6–0.7 cm wide. The weight of 100 inflorescences is 45 g. This cultivar is characterized by a high mass fraction of essential oil (2.0% of the fresh mass).	Total flowering	13 July 2016	2.00
**LI2**	№ 15 (Riabotiagov’s hybrid)	2/136	Shrubs are of large size, semi-spreading shape, 100 cm high, and 110–120 cm in diameter. Elongated cylindrical inflorescences are dense and 10–11 cm long, with 7–8 rings. Corollas are blue. The length of peduncles is 60–65 cm. Leaves are linear gray–green, slightly pubescent, 6.0–6.5 cm long, and 0.7–0.8 cm wide. There are 115-140 flowers in an inflorescence. The fraction of essential oil is about 1.2% of the fresh mass. This cultivar is decorative.	Total flowering	13 July 2016	1.20
**LI3**	Rabat	3/226	Bushes are large and compact: 85–100 cm high and 90–105 cm wide. Inflorescences are dense and 9–11 cm long, with 8–9 rings. Corollas are blue. Leaves are linear gray–green, sparsely pubescent, 5.5–6.0 cm long, and 0.6 cm wide. The weight of 100 inflorescences is 48 g. The mass fraction of essential oil is 1.2% of the fresh mass. This cultivar is hardy.	Total flowering	20 July 2017	1.20
		3/322		Total flowering	02 July 2018	1.20
**LI4**	№ 1-9-16	4/465	Shrubs are large with a semi-spreading shape, 80–100 cm tall, and 90–120 cm in diameter. Inflorescences are cylindrical, dense, 8–10 cm long, and 2.0 cm in diameter, with 6–7 rings. The average number of flowers in a ring is 13. Corollas are blue. Leaves are linear gray–green, slightly pubescent, 4.5–5.5 cm long, and 0.5–0.6 cm wide. The mass fraction of essential oil is 1.2% of the fresh mass. This cultivar is hardy.	Total flowering	08 July 2020	1.20
**LI5**	№ 2-470	5/470	Shrubs are of large size with a semi-spreading shape, a height 90–100 cm, and diameter of 100–120 cm. Inflorescences are cylindrical, dense, and 9–10 cm long. Corollas are blue and moderately pubescent. Leaves are linear gray–green, sparsely pubescent, 5.0–6.0 cm long, and 0.7–0.8 cm wide. The mass fraction of essential oil is 1.2% of the fresh mass. This cultivar is resistant to pests and diseases.	Total flowering	09 July 2020	1.20
**LI6**	Antej	6/472	Shrubs are of large size with a semi-spreading shape, 100 cm high, and 120 cm in diameter. Inflorescences are elongated, cylindrical, dense, large, 11–14 cm long, and 2.5–2.6 cm in diameter, with 7–8 rings. The average ring has from 21 to 27 flowers (average of 24). Corollas are blue. The peduncles are thick and massive. Peduncles are 65–68 cm long. Leaves are linear gray–green, slightly pubescent, 6.5–7.0 cm long, and 0.7–0.8 cm wide. There are 120–170 flowers in inflorescences. The mass fraction of essential oil is 1.7% of the fresh mass. This is an essential oil-bearing cultivar promising for the use of cut flowers. This cultivar is included in the base of the data of the genofund information system of plants in the National Center of Genetic Resources of Plants of Ukraine.	Total flowering	10 July 2020	1.70
**LI7**	№ 1-2-2 (Brovko’s)	7/476	Shrubs are of large size with a semi-spreading shape, 100 cm high, and 110–120 cm in diameter. Inflorescences are cylindrical or spindle-shaped, dense, 9–10 cm long, and 2.2–2.5 in diameter, with 9 rings. The average ring has from 17 to 23 flowers (average 20). Corollas are blue. Peduncles are thick and massive with a length of 65–70 cm. Leaves are linear gray–green, slightly pubescent, 6.0–6.5 cm long, and 0.6–0.7 cm wide. There are 140–150 flowers in inflorescence. The mass fraction of essential oil is 1.4% of the fresh mass. This cultivar is decorative.	Total flowering	13 July 2020	1.40
**LI8**	№ 1-462	16/462	Shrubs are of large size with a semi-spreading shape, 80–100 cm high, and 100–110 cm in diameter. Inflorescences are cylindrical, dense, 9–10 cm long, and 2.0 cm in diameter. Corollas are blue and moderately pubescent. Leaves are linear gray–green, slightly pubescent, 5.5–6.0 cm long, and 0.7 cm wide. The mass fraction of essential oil is 0.9% of the fresh mass. This cultivar is hardy.	Total flowering	07 July 2020	0.90

## Data Availability

Not applicable.

## Supplementary Materials

The following are available online at: https://www.mdpi.com/article/10.3390/molecules27072152/s1, Supplementary Tables S1–S4. Table S1: GC data for the essential oil components identified in samples from different culitvars of L. angustifolia (LA1-7) and L. intermedia (LI1-LI8). Apart from LRI—linear retention indices (experimental and reference values) all figures represents mean of three replicates of % abundance (area percent without solvent peak) *—tentative identification, nd—no data, TF—total flowering; Table S2: Comparative table with % relative abundancies and normative content values given by ISO 3515 (other origins); Table S3: Comparative table with % relative abundancies and normative content values given by Ph. Eur. 10th edition; Table S4: Heatmap of Spearman correlation analysis results. TF—total flowering, EF—end of the total flowering, A and LA—Lavandula angustifolia cultivars, LI—L. intermedia cultivars.

## Sample Availability

Samples of the compounds are not available from the authors.

## Abbreviations

EO, EOs	Essential oil, essential oils
exp.	Experimental
HCA	Hierarchical cluster analysis
LA	*Lavandula angustifolia*
LI	*Lavandula x intermedia*
LL	*Lavandula latifolia*
LRI	Linear retention index
NAAS	National Academy of Agrarian Sciences
nr	Not regulated
PC	Principal component
PCA	Principal component analysis
Ph. Eur.	European Pharmacopeia
SE	Standard error
ref.	Reference
TF	Total flowering
TIC	Total ion current
un	Undetected
